# Supplementary material for: An apicobasal gradient of Rac activity determines protrusion form and position
Source: Nat Commun. 2017 May 19;8:15385. doi: 10.1038/ncomms15385 (PMC5454455; doi:10.1038/ncomms15385)
Supplement: Supplementary Information — Supplementary Figures and Supplementary References [file ncomms15385-s7.pdf]

Supplementary Figure 1

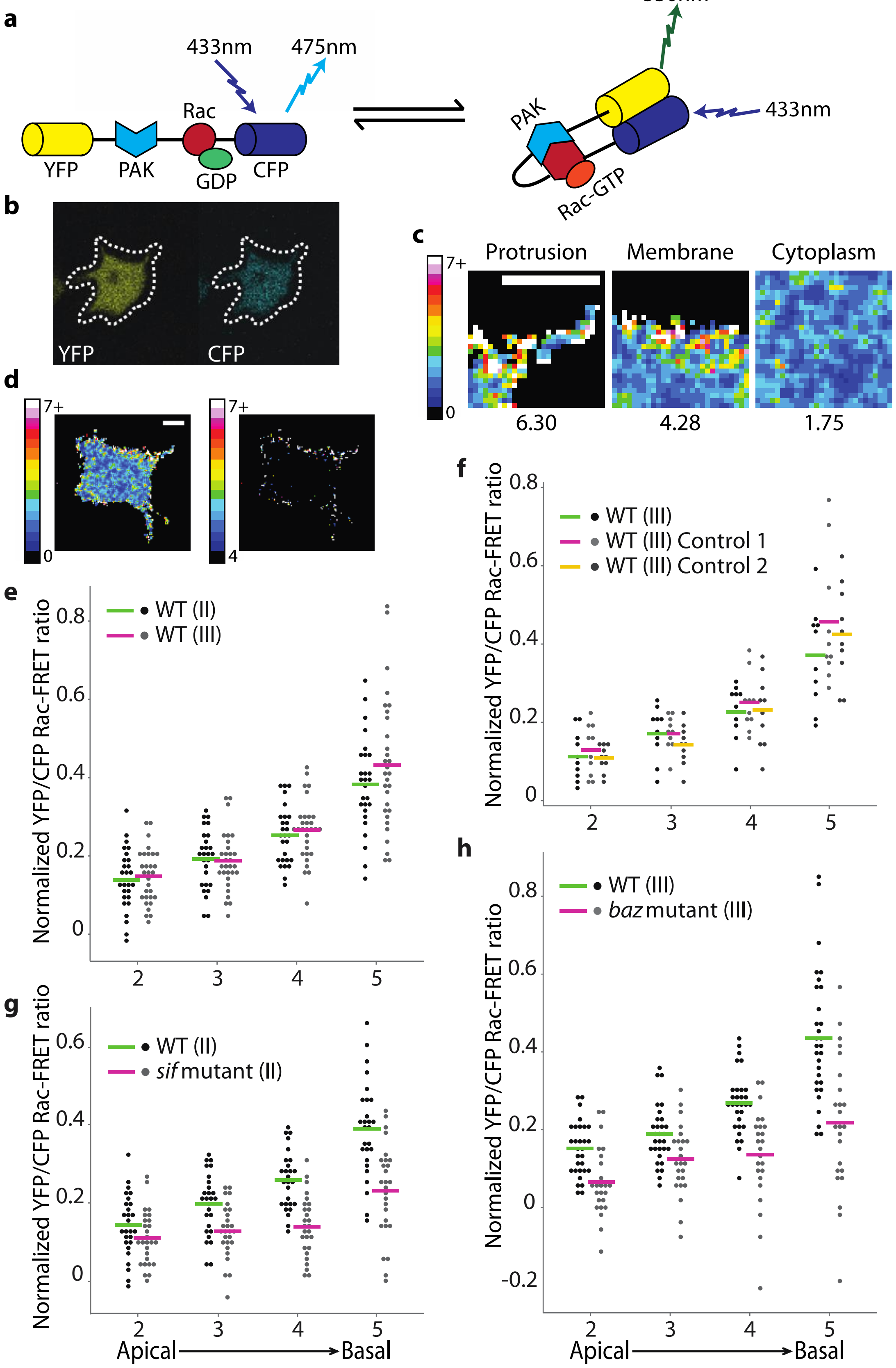

Supplementary Figure 1: FRET construct and control experiments

(A) Schematic representation of the Rac-FRET biosensor before (left) and after (right) activation (adapted from [1]). Active Rac1, bound to GTP, leads to PAK binding, bringing YFP and CFP into close proximity. **(B)** YFP and CFP images at a single *z*-slice of a representative wild-type cell. Dotted lines represent the region used to quantify the mean YFP and CFP signal intensities. **(C-D)** Increased FRET (higher Rac activity) is observed within protrusions and at the cell cortex when compared to within the cytoplasm. (C) Ratiometric FRET images of distinct cellular regions taken from within the same z-slice. The mean FRET ratio within the relevant region of interest is displayed (below images). (D) Comparison of the left-hand image (where all cellular pixels are labelled on the FRET scale 0-7+) with the right-hand image (where only the pixels with high Rac-FRET ratios of 4 and above are labelled) demonstrates that the Rac-FRET probe detects highest Rac activity levels at the plasma membrane. **(E-H)** Dot plots show the normalised YFP/CFP Rac-FRET ratios obtained for each cell analysed, along the apicobasal axis (where 1 is most apical and 5 most basal; see methods for more details). Note the ratio values are normalised to the apical value, making the apical value 0 in all cases, and hence why 1 is not shown on the x-axis. The coloured horizontal bars represent the mean. **(E-F)** Control FRET experiments in wild-type cells. (E) The observed Rac activity gradient is comparable when using a Rac-FRET construct inserted on the 2nd (n=27 cells from four animals) or 3rd chromosome (n=30 cells from ten animals). (F) Different image acquisition methods act as photobleaching controls: Normal acquisition - images acquired from the bottom to the top of the cell, Control 1 - immediate repeat of normal acquisition, and Control 2 - images acquired from the top to the bottom of the cell, (n=10 cells from 3 animals; see methods for further details). **(G-H)** Graphs shown in Figure 1G-H are here represented as Dot plots, which illustrate more clearly inter-cell variation within the sample. Scale bars: 3 µm.

Supplementary Figure 2

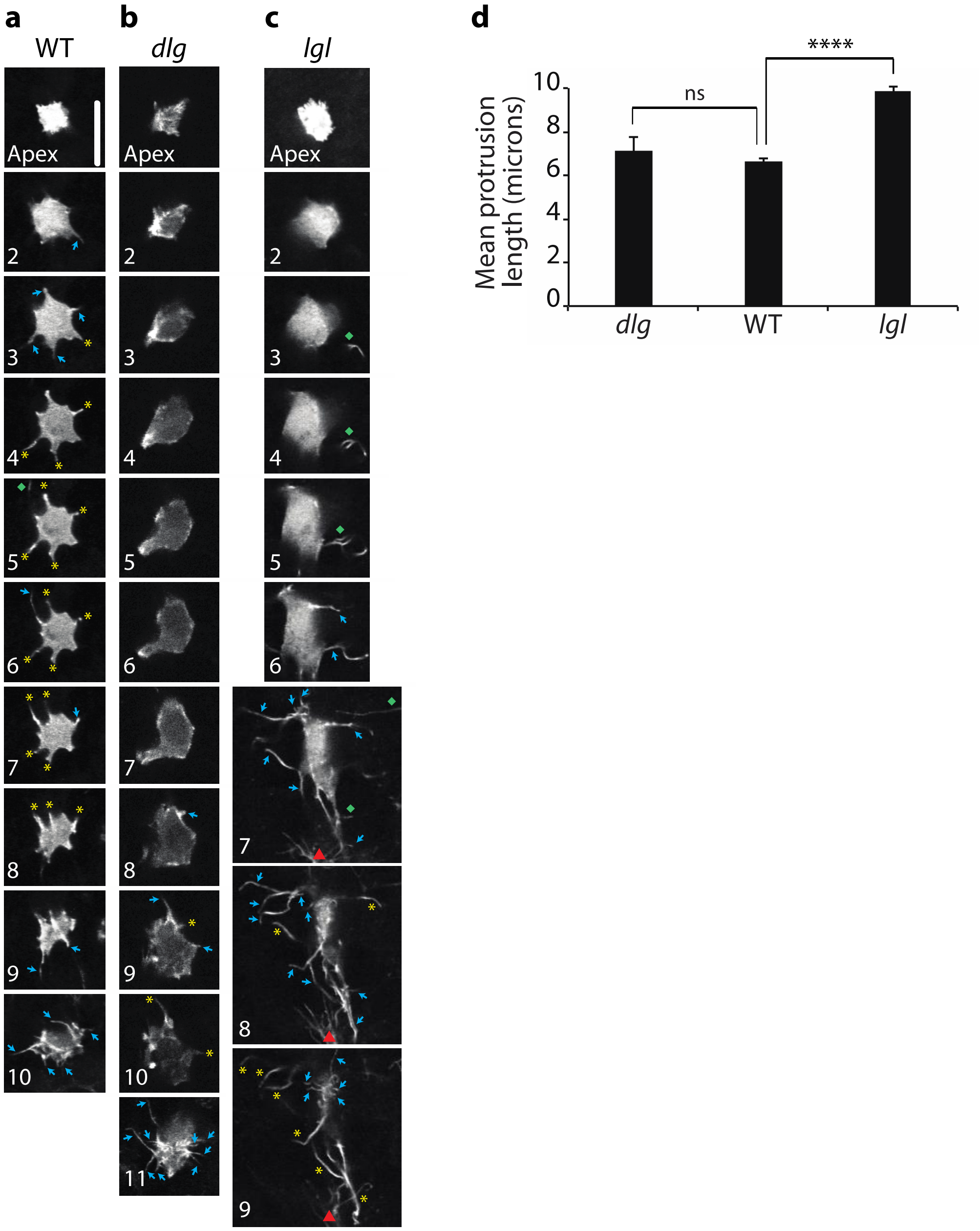

Supplementary Figure 2: *dlg* and *lgl* mutant cell morphologies

(A-C) Live imaging of GFP:Moe-labelled epithelial cells, showing frequent confocal slices from the apex to the base of the cell (numbers represent number of microns from cell apex). Cells shown are: (A) wild-type, (B) *dlg* mutant, (C) *lgl* mutant. Blue arrows show novel protrusions. Yellow asterisks highlight protrusions that were previously seen at a more apical plane; green diamonds, protrusions that originate at a more basal plane. Red triangle shows protrusions from a neighbouring cell. Note how intermediate level protrusions in the wild-type span a number of confocal planes, demonstrating their sheet-like properties (A). Note also the loss of intermediate level protrusions in both *dlg* (B) and *lgl* (C) mutant cells, as well as the highly abnormal basal protrusions in *lgl* mutant cells (C). Scale bar: 10µm. **(D)** Quantification of mean basal protrusion length for *dlg*, wild-type and *lgl* mutant cells. Error bars represent s.e.m. *dlg* n=311 protrusions from 19 animals; wild type n=321 protrusions from 29 animals; *lgl* n=314 protrusions from 17 animals. Student's *t*-test was performed to determine statistical significance and *P* values are shown on graph. $P>0.5$ was considered not-significant (ns), $P<0.05$=*, $P<0.01$=**, $P<0.001$=***, $P<0.0001$=****.

Supplementary Figure 3

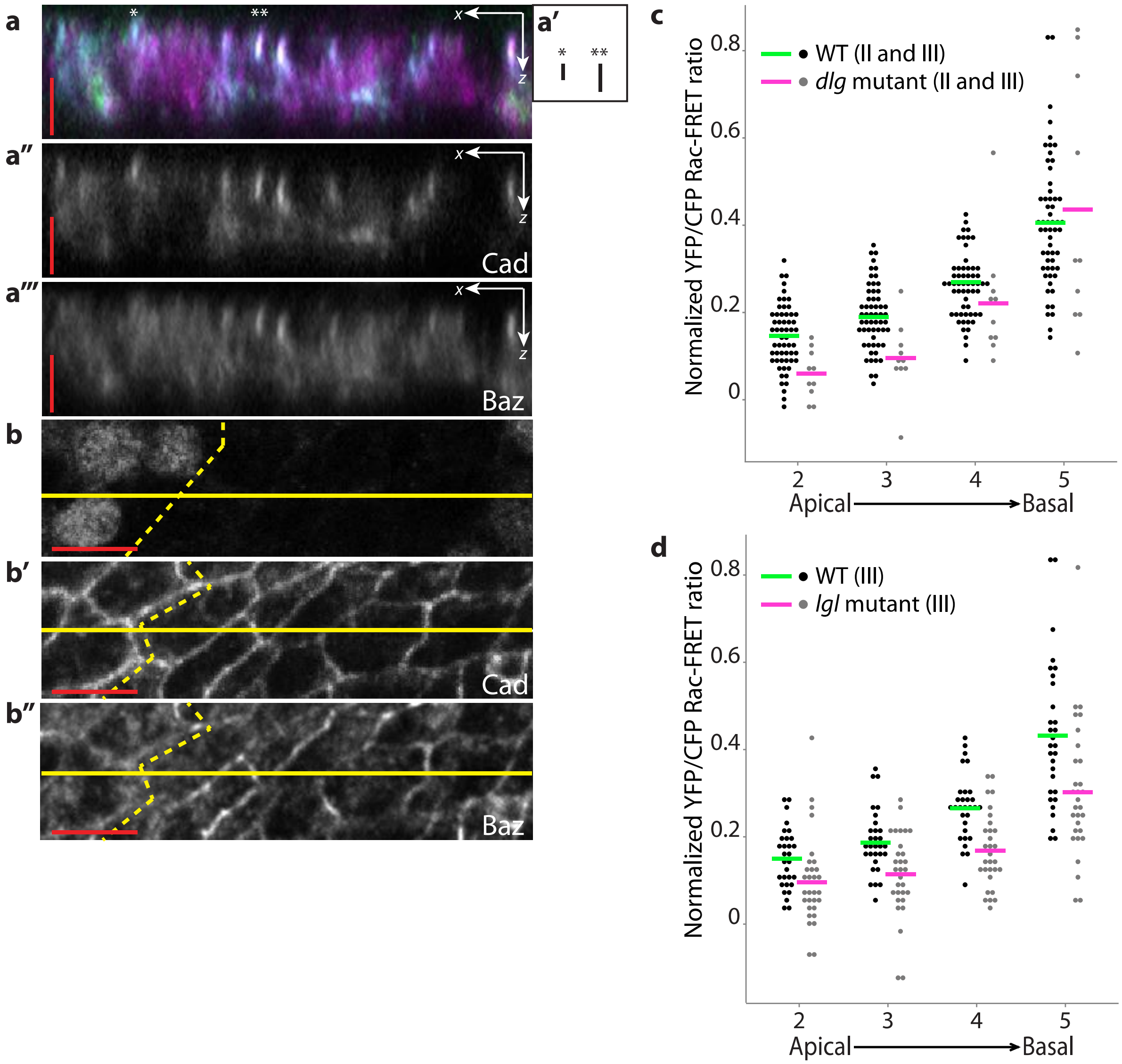

Supplementary Figure 3: Baz colocalises with E-cadherin in expanded junctions.

(A) *x-z* confocal slices illustrating Cad (A and A'') and Baz (A and A''') localisation within fixed and stained mosaic tissue containing *dlg* mutant clones. Note how Baz colocalises with Cad in expanded junctions in the *dlg* mutant clone (see merged panel A). Box (A') highlights expansion of mutant junctions (**) in comparison to neighbouring wild-type junctions (*). **(B-B'')** *x-y* confocal slice; yellow line shows region of *x-z* slice shown in panels A-A'''. A lack of nuclear GFP represents location of homozygous mutant clones (B). Dashed yellow line highlights border between wild type and mutant tissue. Scale bar: 5μm. **(C-D)** Graphs shown in Figure 3E-F are here represented as Dot plots, which illustrate more clearly inter-cell variation within the sample. Plotted are the normalised YFP/CFP Rac-FRET ratios obtained for each cell analysed, along the apicobasal axis (where 1 is most apical and 5 most basal; see methods for more details). Note the ratio values are normalised to the apical value, making the apical value 0 in all cases, and hence why 1 is not shown on the x-axis. The coloured horizontal bars represent the mean.

Supplementary Figure 4

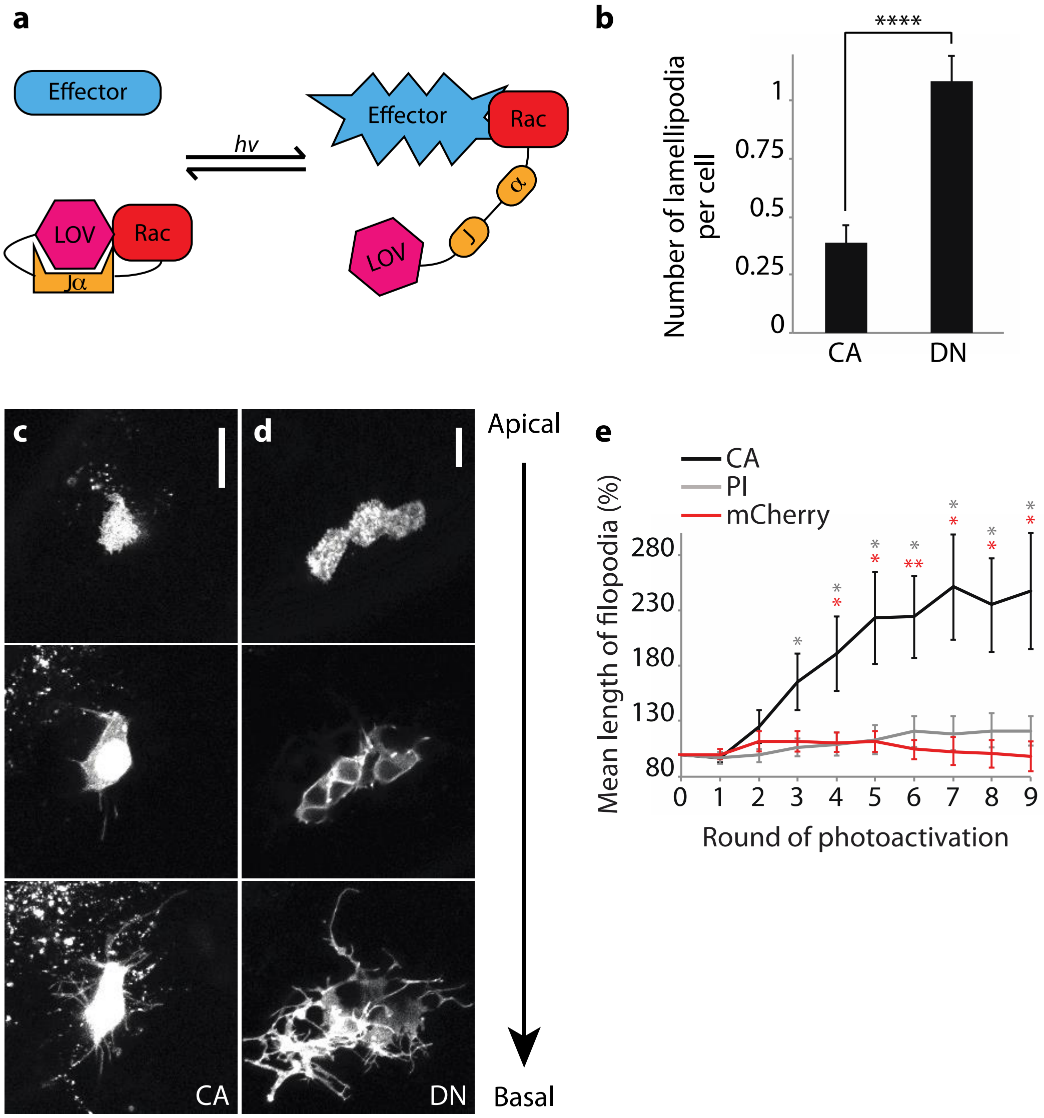

Supplementary Figure 4: Overexpression of photoactivatable Rac constructs

(A) Schematic of the photoactivatable Rac transgenic construct, modified from [2]. In dark conditions, the LOV domain sterically blocks the GTPase active site of Rac. Blue light (hv) causes a change in conformation that results in unwinding of the α helix and exposure of the GTPase domain to downstream Rac effectors. **(B)** Number of basal lamellipodia per cell in cells expressing a constitutively active (CA) form (PA-RacQ61L) and a dominant negative (DN) form (PA-RacT17N) of photoactivatable Rac prior to any experimental photoactivation (n=69 (CA) and 59 (DN) cells). **(C, D)** Representative clones of epithelial cells expressing different forms of PA-Rac prior to any deliberate experimental photoactivation. The different panels show apical, medial and basal *z*-sections to highlight morphological features. **(E)** Quantification of mean filopodial length in cells expressing the constitutively active (CA) form of PA-Rac (black) compared to two different controls: photoinsensitive (PI) PA-Rac (grey) or merely a membrane tethered mCherry construct (red). T-tests compare each control to CA photoactivatable Rac at each time point; n=43 (CA), 39 (PI), 31 (mCherry) filopodia. Error bars represent s.e.m. Scale bars: 10μm. Student's *t*-test was performed to determine statistical significance and *P* values are shown on graph. $P>0.5$ was considered not-significant, $P<0.05$=*, $P<0.01$=**, $P<0.001$=***, $P<0.0001$=****.

Supplementary References

1 Itoh, R. E. *et al.* Activation of rac and cdc42 video imaged by fluorescent resonance energy transfer-based single-molecule probes in the membrane of living cells. *Mol Cell Biol* **22**, 6582-6591 (2002).

2 Wang, X., He, L., Wu, Y. I., Hahn, K. M. & Montell, D. J. Light-mediated activation reveals a key role for Rac in collective guidance of cell movement in vivo. *Nature cell biology* **12**, 591-597, doi:10.1038/ncb2061 (2010).
